# Six SIGMA-based quality assessment of biochemical analytes: A comparative analysis of clinical laboratory improvement amendments 1988 and 2024 standards

**DOI:** 10.5937/jomb0-58564

**Published:** 2025-11-05

**Authors:** Mert Üge, Saliha Aksun

**Affiliations:** 1 Karabuk Public Health Laboratory, Department of Clinical Biochemistry Laboratory, Karabuk, Türkiye; 2 Izmir Katip Celebi University Atatürk Training and Research Hospital, Department of Clinical Biochemistry Laboratory, Izmir, Turkey

**Keywords:** Six SIGMA, quality tool, total allowable error, quality control, Six SIGMA, alat za kvalitet, ukupna dozvoljena greška, kontrola kvaliteta

## Abstract

**Background:**

Laboratories have a responsibility to produce accurate and reliable results to ensure patient safety and meet quality standards. Within the Six SIGMA quality management framework, various approaches may emerge depending on the total allowable error (TAE) limits defined by different standards. This study aimed to compare analyte SIGMA levels using CLIA 1988 and CLIA 2024 criteria, determine appropriate quality control procedures based on Westgard multirules, and identify potential sources of error using the Quality Goal Index.

**Methods:**

SIGMA values for 17 biochemical analytes were calculated based on internal and external quality control data using the formula (TEa - bias)/CV The Quality Goal Index (QGI) was determined using the formula bias/(1.5xCV). All analytes were evaluated at the Karabuk Public Health Laboratory between November 2024 and March 2025.

**Results:**

Amylase (Levels 1 and 2), total bilirubin (Level 1), high-density lipoprotein cholesterol (Levels 1 and 2), creatine kinase (Levels 1 and 2), and lactate dehydrogenase (Levels 1 and 2) demonstrated world-class SIGMA performance according to both CLIA 1988 and CLIA 2024 standards. However, a decline in SIGMA values was observed when calculated using the more stringent CLIA 2024 limits.

**Conclusions:**

The comparison of CLIA 1988 and CLIA 2024 standards demonstrated that stricter TEa limits can significantly impact the performance of SIGMA. While some analytes maintained world-class performance, others exhibited a notable decline, necessitating enhanced quality control measures. These findings emphasise the need for laboratories to periodically reassess test performance in light of evolving regulatory standards to ensure continued analytical reliability and patient safety.

## Abstract (sr)

**Uvod:**

Laboratorije imaju odgovornost da obezbede tačne i pouzdane rezultate kako bi se osigurala bezbednost pacijenata i ispunili standardi kvaliteta. U okviru "Six SIGMA" okvira za upravljanje kvalitetom mogu se pojaviti različiti pristupi u zavisnosti od granica ukupne dozvoljene greške (TAE) koje definišu različiti standardi. Cilj ove studije bio je da se uporede SIGMA nivoi analita korišćenjem kriterijuma CLIA 1988 i CLIA 2024, da se odrede odgovarajuće procedure kontrole kvaliteta na osnovu Westgard pravila, i da se identifikuju potencijalni izvori grešaka korišćenjem Indeksa cilja kvaliteta (QGI).

**Metode:**

SIGMA vrednosti za 17 biohemijskih analita izračunate su na osnovu internih i eksternih podataka kontrole kvaliteta pomoću formule (TEa - pristrasnost)/CV Indeks cilja kvaliteta (QGI) određen je formulom pristrasnost/(1,5xCV). Svi analiti su evaluirani u Zavodu za javno zdravlje Karabuk između novembra 2024. i marta 2025. godine.

**Rezultati:**

Amilaza (nivoi 1 i 2), ukupni bilirubin (nivo 1), holesterol lipoproteina visoke gustine (nivoi 1 i 2), kreatin kinaza (nivoi 1 i 2) i laktat dehidrogenaza (nivoi 1 i 2) pokazali su svetski klasu SIGMA performansi prema standardima CLIA 1988 i CLIA 2024. Međutim, primećen je pad SIGMA vrednosti kada su izračunate prema strožim granicama CLIA 2024.

**Zaključak:**

Poređenje CLIA 1988 i CLIA 2024 standarda pokazalo je da stroža ograničenja ukupne dozvoljene greške mogu značajno uticati na SIGMA performanse. Dok su neki analiti zadržali performanse svetske klase, drugi su pokazali značajan pad, što zahteva pojačane mere kontrole kvaliteta. Ovi nalazi naglašavaju potrebu da laboratorije periodično preispituju performanse testova u svetlu promenljivih regulatornih standarda kako bi se osigurala trajna analitička pouzdanost i bezbednost pacijenata.

## Introduction

Clinical laboratories have an important responsibility to provide reliable and accurate results. Laboratory results may influence the clinician's ability to make an accurate and timely diagnosis, to follow up with the patient and to treat or manage any complications that may arise. Laboratory results not only affect patient safety but also serve as an indicator of the laboratory's quality [1]. Clinical laboratories must execute quality management processes with precision to minimise the risk of analytical errors. Laboratories aim to achieve a high rate of error detection by establishing appropriate quality control procedures for test performance [2]. The preanalytical phase accounts for 60-70% of laboratory errors. However, the implementation of quality studies in the analytical phase is also crucial for evaluating the entire process. Internal and external quality control programmes are essential to minimise analytical error rates [3] [4].

Six Sigma is a process-oriented quality improve ment methodology designed to minimise errors by systematically addressing sources of variation within a system [5]. This methodology consists of three main components: total allowable error (TEa), bias and precision. Six Sigma enables the classification of laboratory tests based on their performance levels, with quality standards ranging from six sigma (world-class) to three sigma (minimum acceptable level) [6]. However, for tests operating at the six sigma level, it is essential to account for random variation around the process target by incorporating a 1.5 standard deviation (SD) shift into the sigma calculation. Failure to do so may lead to significant deviations from the actual performance level [7].

The Quality Goal Index (QGI) serves as a critical parameter within Six Sigma quality management, facilitating the evaluation of analytical test performance by quantifying the relative impact of systematic and random error on total analytical error. When the test performance sigma value is below 5, the cause of poor performance should be determined by calculating the QGI [8].

This study aimed to evaluate the analytical performance of commonly used biochemical analytes within the framework of Six Sigma quality metrics by comparing the impact of two distinct sets of total allowable error limits - those defined by the Clinical Laboratory Improvement Amendments (CLIA) of 1988 and the updated CLIA 2024 guidelines. The study further aimed to explore how changes in these limits influence quality planning practices in clinical laboratories.

## Materials and methods

A total of 17 biochemical parameters including aspartate aminotransferase (AST), alanine aminotransferase (ALT), amylase, alkaline phosphatase (ALP), gamma-glutamyl transferase (GGT), glucose, albumin, total protein, total bilirubin, calcium, total cholesterol, triglycerides, high-density lipoprotein cholesterol (HDL), creatine kinase (CK), creatinine, phosphorus, and lactate dehydrogenase (LDH)-were analysed using the Roche Cobas c501 analyser (Roche Diagnostics GmbH, Mannheim, Germany).

The coefficient of variation (CV%) was calculated based on internal quality control (IQC) data collected at two concentration levels for each analyte between November 2024 and March 2025 in our laboratory. Internal quality control analyses were conducted daily over five months using PreciControl ClinChem Multi 1 (lot no. 70496600) and Multi 2 (lot no. 70499200) as Level 1 and Level 2 quality control materials, respectively (Roche Diagnostics GmbH, Germany). Bias was determined through participation in an external quality control (EQC) program (KBUDEK, Turkey), conducted between November 2024 and March 2025, involving laboratories utilising the same analytical method. EQC material was run once a month every month.

The formula used for CV% and bias calculation is as follows.

CV (%) = (SD/mean) x 100

Bias = [(laboratory results)-(mean of peer group)/(mean of peer group)] x 100

Sigma metrics were calculated based on total allowable error limits defined by the CLIA'88 and CLIA'24. Sigma metrics were evaluated according to their performance as follows: σ≥6 world-class, 5≤σ<6 excellent, 4≤σ<5 good, 3≤σ<4 marginal, 2≤σ<3 poor, σ<2 unacceptable [9] [10].

Sigma = (TEa-bias)/CV

QGI was determined for parameters with sigma values below 5 using CV% and bias.

QGI = Bias/(CV x 1.5)

The problem behind the parameters that perform poorly according to sigma quality is determined as follows: QGI < 0.8 imprecision, QGI > 1.2 inaccuracy, QGI between 0.8 and 1.2 both imprecision and inaccuracy [6].

The calculations (CV, bias, Sigma, and QGI) were performed using the Microsoft Excel programme (Microsoft Corporation, Redmond, WA; Excel 2010). Ethical approval for the study was obtained from the Ethics Committee of Karabuk University (Application No: 2025/2228) on April 11, 2025.

## Results

Bias and coefficient of variation (CV) values for all analytes at both control levels were calculated over five months (Table 1).

**Table 1:** Coefficient of variation and bias values of analytes.

Analytes	CV_Level 1_	CV_Level 2_	Bias
AST	3.06	2.12	-7.2
ALT	1.96	2.36	-9.6
Amylase	2.65	2.61	1.4
ALP	3.30	2.49	-3.4
GGT	2.22	2.00	8.7
Glucose	2.24	2.07	-2
Albumin	2.67	2.13	-5.4
Total protein	1.75	2.11	2.5
Total bilirubin	2.52	4.72	-1.6
Calcium	2.81	2.12	-2.3
Total cholesterol	3.09	2.97	-6.7
Triglyceride	3.25	2.92	3.9
HDL	2.65	2.10	-2.3
CK	2.28	2.45	-0.3
Creatinine	3.58	4.25	2.1
Phosphorus	1.77	3.14	3.8
LDH	1.40	1.34	-2.3

For the analytes' total bilirubin, calcium, total cholesterol, and phosphorus, the total allowable error (TEa) criteria remained unchanged between the ClIA'88 and CLIA'24 standards. Total bilirubin (L1) exhibited world-class performance (σ≥6), while total bilirubin (L2), calcium (L1), and phosphorus (L1) have shown marginal performance (3≤σ<4). Total cholesterol (L1 and L2) and phosphorus (L2) demonstrated unacceptable performance (σ<2). Amylase, HDL, CK, and LDH exhibited world-class sigma performance (σ≥6) at both levels (L1 and L2) according to CLIA'88 and CLIA'24 total allowable error criteria (Table 2).

**Table 2:** Comparison of analyte sigma values according to CLIA'88 and CLIA'24.

Analytes	TEa Sources		SigmaLevel 1	SigmaLevel 2
AST	CLIA'88	20	4.18	6.03
	CLIA'24	15	2.46	3.67
ALT	CLIA'88	20	5.30	4.40
	CLIA'24	15	2.75	2.28
Amylase	CLIA'88	30	10.79	10.95
	CLIA'24	20	7.01	7.12
ALP	CLIA'88	30	8.06	10.68
	CLIA'24	20	5.03	6.66
GGT	CLIA'88	20	5.09	5.65
	CLIA'24	15	2.83	3.15
Glucose	CLIA'88	10	3.57	3.86
	CLIA'24	8	2.67	2.89
Albumin	CLIA'88	10	1.72	2.15
	CLIA'24	8	0.97	1.22
Total protein	CLIA'88	10	4.28	3.55
	CLIA'24	8	3.14	2.60
Total bilirubin	CLIA'88	20	7.30	3.89
	CLIA'24	20	7.30	3.89
Calcium	CLIA'88	11	3.09	4.10
	CLIA'24	11	3.09	4.10
Total cholesterol	CLIA'88	10	1.06	1.11
	CLIA'24	10	1.06	1.11
Triglyceride	CLIA'88	25	6.49	7.22
	CLIA'24	15	3.41	3.80
HDL	CLIA'88	30	10.45	13.19
	CLIA'24	20	6.67	8.42
CK	CLIA'88	30	13.02	12.12
	CLIA'24	20	8.64	8.04
Creatinine	CLIA'88	15	3.60	3.03
	CLIA'24	10	2.20	1.85
Phosphorus	CLIA'88	10	3.50	1.97
	CLIA'24	10	3.50	1.97
LDH	CLIA'88	20	12.64	13.20
	CLIA'24	15	9.0	9.47

According to CLIA'88 standards, analytes demonstrating world-class sigma performance (ρ≥6) included AST (L2), ALP (L1 and L2), and triglycerides (L1 and L2). Those with excellent performance (5≤σ<6) were ALT (L1) and GGT (L1 and L2). Good performance (4≤σ<5) was observed for AST (L1), ALT (L2), and total protein (L1). Glucose (L1 and L2), total protein (L2), and creatinine (L1 and L2) have shown marginal performance (3≤σ<4). Albumin (L2) exhibited poor performance (2≤σ<3), while albumin (L1) showed unacceptable performance (σ<2) (Table 2).

According to CLIA 2024 standards, ALP (L2) demonstrated world-class sigma performance (σ≥6), while ALP (L1) showed excellent performance (5≤σ<6). Marginal performance (3≤σ<4) was observed for AST (L2), GGT (L2), total protein (L1), and triglycerides (L1 and L2). Poor sigma performance (2≤σ<3) was observed for AST (L1), ALT (L1 and L2), GGT (L1), glucose (L1 and L2), total protein (L2), and creatinine (L1). Albumin (L1 and L2) and creatinine (L2) have shown unacceptable performance (σ<2) (Table 2).

QGI was determined to identify the cause of the problem in analytes with Sigma levels below 5 at both control levels. Analytes with a QGI > 1.2, indicating an inaccuracy problem at both levels (L1 and L2), were AST, ALT, GGT, albumin and total cholesterol. Parameters with a QGI<0.8, indicating an imprecision problem at both levels (L1 and L2), were glucose, calcium, and creatinine (Table 3).

**Table 3:** Calculated quality goal index for analytes and sources of the problem.

Analytes	QGILevel 1	QGILevel 2	Problem
AST	1.56	2.26	inaccuracy (L1 and L2)
ALT	3.26	2.71	inaccuracy (L1 and L2)
Amylase	-	-	None
ALP	-	-	None
GGT	2.61	2.91	inaccuracy (L1 and L2)
Glucose	0.59	0.64	imprecision (L1 and L2)
Albumin	1.35	1.68	inaccuracy (L1 and L2)
Total protein	0.95	0.79	inaccuracy and imprecision (L1), imprecision (L2)
Total bilirubin	**-**	0.22	imprecision (L2)
Calcium	0.54	0.72	imprecision (L1 and L2)
Total cholesterol	1.44	1.50	inaccuracy (L1 and L2)
Triglyceride	0.81	0.89	inaccuracy and imprecision (L1 and L2)
HDL	-	-	None
CK	-	-	None
Creatinine	0.39	0.32	imprecision (L1 and L2)
Phosphorus	1.43	0.81	inaccuracy (L1), inaccuracy and imprecision (L2)
LDH	-	-	None

Appropriate quality control procedures were demonstrated for analytes with a Six Sigma scale range calculated between 3 and 6 (Table 4).

**Table 4:** Analyte-specific QC procedures according to six sigma performance.

Sigma<br>Range	Analytes with sigma value <br>calculated according to CLIA'88	Analytes with sigma value <br>calculated according to CLIA'24	QC procedures	Run
σ≥6	Amylase (L1 and L2), ALP (L1 and L2), triglyceride (L1 and L2), HDL (L1 and l2), CK (L1 and L2), LDH (L1 and L2), total bilirubin (L1), AST (L2)	Amylase (L1 and L2), ALP (L2), HDL (L1 and L2), CK (L1 and L2), LDH (L1 and L2), total bilirubin (L1)	1_3s_	N = 2 <br>R=1
5≤σ<6	ALT( L1), GGT (L1 and L2)	ALP (L1)	1_3s_/2_2s_/R_4s_	N = 2 <br>R=1
4≤σ<5	AST (L1), ALT (L2), total protein (L1), calcium (L2)	Calcium (L2)	1_3s_/2_2s_/R_4s_/R_1s_	N=4 or <br>N=2 <br>R=1 <br>R=2
3≤σ<4	glucose (L1 and L2), total protein (L2) total bilirubin (L2), calcium (L1), creatinine (L1 and L2), phosphorus (L1)	AST (L2), GGT (L2), total protein (L1), total bilirubin (L2), calcium (L1), triglyceride (L1 and L2), phosphorus(L1)	1_3s_/2_2s_/R_4s_/4_1s_/8_x_	N=4 or <br>N = 2 <br>R=2 <br>R=4

## Discussion

**Figure 1:**
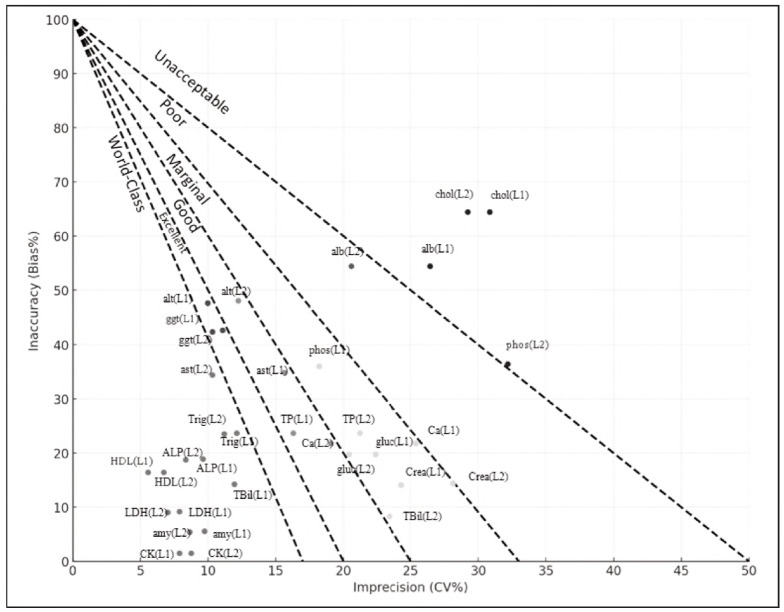
Graphical method decision chart depicting analytical performance of 17 biochemical parameters based on CLIA 1988 TEa criteria.

**Figure 2:**
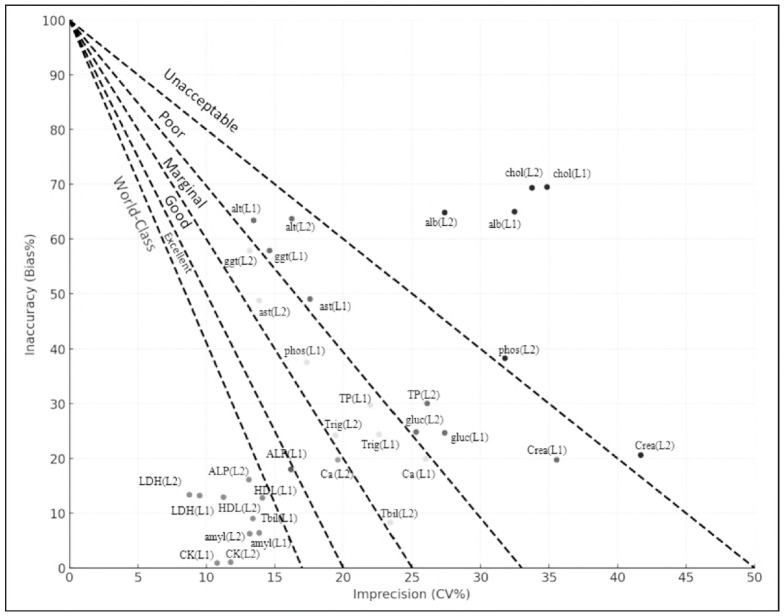
Graphical method decision chart depicting analytical performance of 17 biochemical parameters based on CLIA 2024 TEa criteria.

In clinical laboratory settings, the Sigma methodology is employed as a systematic quality improvement approach to monitor analytical performance and identify errors associated with both precision and accuracy [5]. Using the Six Sigma methodology, this study evaluated the analytical performance of 17 routine biochemical analytes based on total allowable error limits defined by the CLIA 1988 and the updated CLIA 2024 guidelines. The results, including the components of imprecision and inaccuracy, are illustrated in the method decision charts presented in Figure 1 and Figure 2. Except for total bilirubin, calcium, total cholesterol, and phosphorus, the CLIA 2024 guidelines have introduced more stringent limits for the remaining biochemical parameters.

Total bilirubin at Level 1 demonstrated a world-class sigma performance (σ≥6), which may be attributed to its relatively low mean CV and bias observed over the five-month assessment period. On the other hand, total cholesterol at both levels and phosphorus at Level 2 demonstrated inadequate sigma performance (σ<2), not meeting the minimum acceptability criteria (Table 2). We believe this may be due to the strict TEa limits set for these tests, combined with the higher bias values observed compared to other analytes. Kumar BV Mahan T. [11] evaluated the analytical performance of several biochemical parameters using Six Sigma metrics and, similar to our results, found that total cholesterol did not meet the acceptable sigma performance criteria. The Sigma metrics were calculated as 1.51 at Level 1 and 1.83 at Level 2 [11].

In our study, AST (Level 1), ALT (Levels 1 and 2), GGT (Level 1), glucose (Levels 1 and 2), total protein (Level 2), and creatinine (Level 1) exhibited poor sigma performance (2≤σ<3) according to CLIA 2024 criteria, with values remaining close to the minimum acceptable limits. We believe that these tests should be followed more closely in the quality monitoring process after the implementation of the CLIA 2024 standards. Similar to our findings, a study conducted by Gadde et al. [12] using the Cobas 6000 analyser reported sigma values for creatinine of 2.5 at Level 1 and 2.3 at Level 2, indicating poor analytical performance. In our study, despite the more stringent TEa limits introduced by CLIA 2024, amylase, HDL, CK, and LDH demonstrated world-class sigma performance at both quality control levels. Similar to our findings, Singh et al. reported high sigma values for amylase (11.2 at Level 2 and 11.7 at Level 3), CK (8.3 at Level 2 and 9.0 at Level 3), and HDL (6.3 at Level 2), all indicating world-class analytical performance [13].

In our study, following the transition from CLIA1988 to CLIA 2024, a notable decline in sigma performance was observed for AST at Level 1, ALT at Levels 1 and 2, GGT at Level 1, and triglycerides at Levels 1 and 2. There are limited studies in the current literature that compare the sigma performance of biochemical parameters based on CLIA standards (Table 4). In a study conducted by Karam et al. at Mansoura University Children's Hospital, the sigma metrics of 14 biochemical parameters were evaluated, and the CLIA'88 and CLIA 2024 total allowable error limits were compared, similar to our study. In their research, only BUN at Levels 1 and 2 exhibited sigma performance below 3 when evaluated using CLIA'88 limits. However, when evaluated using the updated CLIA 2024 criteria, additional parameters - including creatinine, glucose at Level 1, and creatinine at Level 2 - were also found to have sigma values below 3 [14]. In another study, Kele et al. observed that although the majority of 21 routine biochemistry parameters demonstrated good performance according to CLIA criteria, their sigma metrics failed to meet even the acceptable performance limits when evaluated against biological variation (BV)-based goals [15].

When QGI was calculated, AST, ALT, GGT, albumin, and total cholesterol showed values above 1.2 at both levels (Table 3). We interpreted this result as an expression of a systematic error. Systematic errors refer to a consistent deviation that affects all measurements in the same direction. They are attributed to calibration inaccuracies, reagent lot changes, instrument malfunction and methodological biases. If unre cognised, they can lead to inaccurate test results and misinterpretations. To minimise the impact of systematic errors, corrective actions such as recalibrating analytical instruments, verifying the integrity of reagent lots, and revalidating methods should be implemented. The QGI for glucose, calcium and creatinine tests were below 0.8 at both levels (Table 3). Therefore, we thought it would be beneficial to examine random error sources related to precision. Random errors refer to unpredictable errors. They are attributed to reasons such as pipetting errors, envi ronmental conditions (including electrical and tempe ature fluctuations), and inconsistencies in sample handling. In the case of random errors, precision can be improved through enhanced staff training on pipetting techniques, stringent control of environmental conditions (e.g., temperature and voltage sta bility), and reinforcement of standardised sample handling protocols.

Amylase (Levels 1 and 2), ALP (Level 2), total bilirubin (Level 1), HDL (Levels 1 and 2), CK (Levels 1 and 2), and LDH (Levels 1 and 2) demonstrated sigma values of ≥ 6 based on CLIA 2024 limits (Table 4). In light of these findings, the 1_3s_ rule was considered an appropriate quality control protocol among the Westgard multirule strategies for these analytes. On the other hand, AST (Level 2), GGT (Level 2), total protein (Level 1), total bilirubin (Level 2), calcium (Level 1), triglycerides (Levels 1 and 2), and phosphorus (Level 1) exhibited sigma performance within the range of 3≤σ<4 (Table 4). Therefore, these analytes should be monitored using a Westgard multirule 1_3s_/2_2s_/R_4s_/4_1s_/8_x_. Westgard Sigma Rules provide a straightforward and efficient method for selecting the number of control measurements and control rules to detect significant errors. It demonstrates its applicability in clinical laboratories for analytes with low standard deviations (ρvalues) [16].

Analytes demonstrating world-class sigma performance can enhance diagnostic accuracy, reduce the likelihood of inappropriate treatments, and help avoid unnecessary repeat testing in clinical practice. Conversely, for underperforming analytes, laboratory professionals should implement corrective actions based on the identified source of error. It should also be considered that errors not only in the analytical phase but also in the preanalytical and post-analytical phases should be examined.

The limitations of our study include its single-centre design, restriction to a specific analyser and reagent brand, reliance on CLIA guidelines for TEa limits, and a limited number of external quality control cycles. Future research should include multicenter evaluations and compare TEa limits established by various sources (e.g., Rilibak, RCPA, BV) to further validate and expand upon the present findings.

## Conclusions

In this study, the analytical performance of 17 routine biochemical analytes was evaluated across two internal quality control levels using the Six Sigma methodology, based on total allowable error limits defined by the Clinical Laboratory Improvement Amendments (CLIA) of 1988 and 2024. Sigma metrics, along with the coefficient of variation (CV), bias, and Quality Goal Index (QGI), were calculated and summarised to identify analytes with imprecision and/or inaccuracy problems. Our findings revealed notable differences in sigma performance between the two CLIA standards, emphasising the impact of updated TEa limits on quality assessment. Based on the classification of sigma levels, appropriate quality control procedures were recommended using Westgard multirule strategies. These results underscore the importance of analyte performance evaluation and tailored quality control (QC) planning in clinical laboratories to ensure test reliability and patient safety.

## Dodatak

### Acknowledgements

We would like to express our sincere gratitude to the healthcare professionals working in our laboratory for their valuable technical assistance.

### Conflict of interest statement

All the authors declare that they have no conflict of interest in this work.

### List of abbreviations

ALP alkaline phosphatase;<br>ALT, alanine aminotransferase;<br>AST, aspartate aminotransferase;<br>CK, crea tine kinase;<br>CLIA, Clinical Laboratory Improvement Amend ments;<br>CV, coefficient of variation;<br>EQC, external quality control;<br>GGT, gamma-glutamyl transferase;<br>HDL, high-density lipopro tein cholesterol;<br>LDH, lactate dehydrogenase;<br>IQC, internal quality control;<br>SD, standard deviation;<br>QGI, Quality Goal Index;<br>TEa, total allowable error
